# Treatment of *Plasmodium chabaudi* Parasites with Curcumin in Combination with Antimalarial Drugs: Drug Interactions and Implications on the Ubiquitin/Proteasome System

**DOI:** 10.1155/2013/429736

**Published:** 2013-04-03

**Authors:** Zoraima Neto, Marta Machado, Ana Lindeza, Virgílio do Rosário, Marcos L. Gazarini, Dinora Lopes

**Affiliations:** ^1^Unidade de Parasitologia, Instituto de Higiene e Medicina Tropical (IHMT), Universidade Nova de Lisboa, Rua da Junqueira 100, 1349-008 Lisbon, Portugal; ^2^Centro de Malária e Doenças Tropicais (CMDT), Instituto de Higiene e Medicina Tropical (IHMT), Universidade Nova de Lisboa, Rua da Junqueira 100, 1349-008 Lisbon, Portugal; ^3^Departamento de Biociências, Universidade Federal de São Paulo, Avenida Ana Costa 95, 11060-001 Santos, SP, Brazil

## Abstract

Antimalarial drug resistance remains a major obstacle in malaria control. Evidence from Southeast Asia shows that resistance to artemisinin combination therapy (ACT) is inevitable. Ethnopharmacological studies have confirmed the efficacy of curcumin against *Plasmodium* spp. Drug interaction assays between curcumin/piperine/chloroquine and curcumin/piperine/artemisinin combinations and the potential of drug treatment to interfere with the ubiquitin proteasome system (UPS) were analyzed. *In vivo* efficacy of curcumin was studied in BALB/c mice infected with *Plasmodium chabaudi* clones resistant to chloroquine and artemisinin, and drug interactions were analyzed by isobolograms. Subtherapeutic doses of curcumin, chloroquine, and artemisinin were administered to mice, and mRNA was collected following treatment for RT-PCR analysis of genes encoding deubiquitylating enzymes (DUBs). Curcumin was found be nontoxic in BALB/c mice. The combination of curcumin/chloroquine/piperine reduced parasitemia to 37% seven days after treatment versus the control group's 65%, and an additive interaction was revealed. Curcumin/piperine/artemisinin combination did not show a favorable drug interaction in this murine model of malaria. Treatment of mice with subtherapeutic doses of the drugs resulted in a transient increase in genes encoding DUBs indicating UPS interference. If curcumin is to join the arsenal of available antimalarial drugs, future studies exploring suitable drug partners would be of interest.

## 1. Introduction

Malaria remains a major cause of mortality and morbidity especially in sub-Saharan Africa. Children under five and pregnant women remain the most vulnerable groups afflicted by this disease [1]. Control programs are strongly affected by resistance to insecticides and to antimalarials, including the recently implemented combination therapies with artemisinin and its derivatives [2, 3]. Development of new antimalarial drugs is necessary though expensive and time consuming, and a number of potential antimalarials exist either derived from plants or as new synthetic compounds [4]. Parasite drug-resistance and its spread throughout the world can develop quite rapidly (as exemplified by the drug pyrimethamine/sulfadoxine) [4], and there is a need to intensify the search for new antimalarial agents preferably acting on newer targets. Curcumin, the active compound derived from the plant *Curcuma longa*, has anticancer, anti-inflammatory, antiviral, and antimalarial activity [5–10]. Curcumin has also shown potent activity against other organisms including: *Schistosoma mansoni* adult worms, *Cryptosporidium parvum*, and *Trypanosoma cruzi* [8–10].

Previous studies have shown that a combination of oral curcumin and intramuscular administration of artemisinin derivative *α*-*β* arteether to *P. berghei*-infected mice improved the survival rates and prevented recrudescence [6]. In contrast, a previous work done by our group [7] has shown that oral administration of 300 mg/kg of body weight (bw) of curcumin in combination with 20 mg/kg/bw of piperine and 150 mg/kg of artemisinin showed no conclusive effect on the course of infection [7]. However, antimalarial activity and peak parasitemia reached by the curcumin and curcumin/piperine treatment groups were significantly lower compared to the control untreated group [7].

It is not clear what the targets of curcumin are. Some authors have suggested that the target of curcumin might be similar to that which initially was thought to be the target of artemisinin (*PfATP6*) [11]; however, a recent study has shown that the targets of artemisinin remain unclear [12]. Others have shown that curcumin might be an inhibitor of histone acetyltransferase (HAT) and it can also induce the production of reactive oxygen species which may contribute to parasite's death [13]. However, a recent study has shown that curcumin may interfere with many signaling pathways [14] including: the mitogen-activated protein kinases (MAPKs), casein kinase II (CKII), and the COP9 signalosome (CSN) as well as the ubiquitin proteosome pathway (UPS) which will be analyzed in this study [14].

An *in silico* study [15] has shown that the genome of *Plasmodium* spp. contains several genes encoding proteins predicted to be involved in the UPS [15]. Ubiquitylation is a regulated posttranslational modification of proteins in which an ubiquitin molecule is attached to a lysine amino acid in the target protein [15, 16]. Attachment of ubiquitin molecules to proteins is catalyzed by the action of ubiquitin activating enzymes (E1), ubiquitin-conjugating enzyme (E2), and ubiquitin ligase (E3) [15, 16]. In general, ubiquitylation linked via Lys29 or Lys48 with four or more ubiquitin molecules is targeted for degradation by the proteasome [17, 18]. On the other hand, ubiquitylation linked via Lys63 on the target protein is involved in the regulation of a myriad of cellular processes [17, 18].

The removal of ubiquitin molecules is carried out by de-ubiquitylating enzymes (DUBs) [15–19], which are responsible for the generation of free ubiquitin molecules and the disassembly of mono- or polyubiquitin chains on substrate proteins [15–19]. The *Plasmodium* genome encodes at least 20 to 40 putative DUBs [15–19] which are classified as cysteine proteases and zinc-dependent metalloproteases based on their ubiquitin protease domain [15–19]; these are: the ubiquitin C terminal hydrolases (UCHs), the ubiquitin specific proteases (USP/UBPs), the Machado—Joseph disease protein domain proteases (MJDs), the Otubains (OTUs), the JAMM motif metalloproteases (JAMMs), and the permuted papain fold peptidase (PPPDE) as well as [15–19] deubiquitylating-like enzymes (DUBLs), which have been thoroughly reviewed by others [15–19].

 It has become apparent that deubiquitylation plays an important role in the regulation of the UPS as confirmed by aberrations in genes encoding DUBs [20]. Furthermore, DUBs have also been implicated in antimalarial drug resistance as confirmed by mutations found in a gene encoding a de-ubiquitylating enzyme UBP-1 (MAL1P1.34b) in *Plasmodium chabaudi* parasites resistant to artemisinin and artesunate [21]. The V2697F and V2728F mutations lie close to the catalytic site of the enzyme and probably affect protein structure and function [21]. However, the role of those mutations in artemisinin drug resistance is yet to be clarified through transfection assays [21]. Taken together this data shows that the UPS represents a promising antimalarial drug target [22].

The aim of the present study was to analyze the efficacy and the drug interactions between curcucmin/piperine/chloroquine and curcumin/piperine/artemisinin in *Plasmodium chabaudi* parasites resistant to chloroquine (AS-3CQ) and artemisinin (AS-ART) and to verify the effects of curcumin, chloroquine, and artemisinin drug treatment on the UPS.

## 2. Materials and Method

### 2.1. *Plasmodium chabaudi* Parasite Clones

*Plasmodium chabaudi* clones available in our database and used in this study were AS-3CQ (resistant to chloroquine) and selected from the clone AS-Pyr which was subjected to six daily doses of chloroquine (CQ) at 3 mg/kg [23]. This parasite line was cloned and named AS-3CQ [23]. The AS-ART clone resistant to artemisinin was obtained from a clone known as AS-30CQ which tolerated 300 mg/kg/day of artemisinin [24] obtained by serial passages in the presence of increasing subcurative doses of artemisinin [23, 24]; this parasitic line was cloned and named AS-ART [24]. The clones displayed a stable phenotype even after freezing/thawing serial blood passages through mice in the absence or presence of drug treatment, and transmission through the mosquito vector *Anopheles stephensi*.

### 2.2. Acute Toxicity of Curcumin

BALB/c male mice weighing 15 g and 6-7 weeks old were purchased from the animal house facility at the IHMT (Institute of Hygiene & Tropical Medicine, Lisbon, Portugal). The LD50 of curcumin in BALB/c mice was determined by oral administration of five doses 2 g/kg/bw; 2,5 g/kg/bw; 3 g/kg/bw; 3,5 g/kg/bw; and 5 g/kg/bw to each individual mouse after four hours of fasting. Five grams is the concentration reported by others [27, 28] to be the highest dose known to be administered to mice for the acute toxicity test of any drug. Animals were observed for 14 days for any physical signs of toxicity including trembling, lethargic behavior, and impaired body movements.

### 2.3. *In Vivo* Four-Day Suppressive Test of Curcumin, Curcumin/Piperine, Curcumin/Piperine/Chloroquine, and Curcumin/Piperine/Artemisinin

In the present study the *in vivo* efficacy and the interaction of curcumin/piperine in combination with artemisinin and chloroquine was assayed using the 4-day suppressive test [29]. Curcumin 94% cucuminoid content (Sigma-Aldrich, Madrid, Spain) and artemisinin (Sigma-Aldrich, Madrid, Spain) were dissolved in DMSO (Sigma-Aldrich, Madrid, Spain) and chloroquine (Sigma-Aldrich, Madrid, Spain) was dissolved in water. The parasites kept in liquid nitrogen were thawed and mice were inoculated with 1 × 10^6^ infected red blood cells. Parasitemia was allowed to evolve and once parasitemia reached 30%, infected blood was collected and diluted with citrate saline solution.

An intraperitoneal injection of 1 × 10^6^ infected red blood cells was administered to individual mice. Cages contained a maximum of 5 mice each and were kept in a light-dark cycle and mice had food and water *ad libitum*. Three hours later mice were administered by oral gavage either chloroquine alone, curcumin alone, or the combination of curcumin/piperine/chloroquine as piperine 97% (Sigma-Aldrich, Madrid, Spain) has been reported in a previous study to have no antimalarial activity [7], but it is reported to enhance curcumin uptake [30]. 

The same procedure was carried out in mice infected with AS-ART resistant parasites. Infected mice received an inoculum of 1 × 10^6^ infected red blood cells and three hours later groups of 5 mice per cage were administered an oral dose of artemisinin alone, curcumin alone, and curcumin/piperine, and another group received a combination of curcumin/piperine/artemisinin. All experiments included a drug-free control group. Drugs were administered orally for 4 days (Day 0, 1, 2, 3). Parasitemia was monitored every day following drug treatment for a period of 7 consecutive days, as previous work has shown that differences in curcumin/piperine combination versus the control group can be observed between 5 and 7 days after drug treatment had ended [7]. Thin blood smears were prepared and stained with 20% Giemsa/PBS solution (Sigma-Aldrich, Madrid, Spain) pH 7.2, and microscopic slides were analyzed by light microscopy. 

### 2.4. *In Vivo* Drug Interaction Studies and Isobolograms

The ED50 values (the concentration that produces 50% reduction of parasitemia) of the drug alone and in combination were calculated by plotting the log dose versus relative percentage inhibition using GraphPad Prism 4 Software (GraphPad Prism 4, CA, USA) using nonlinear regression, dose-response curve according to the 4-parameter logistic equation (Hill slope). From the ED50 and the Hill slope the ED90 values were calculated using the formula LogED50 = LogED90 − (1/Hill  Slope) × log⁡(9) and the equation *Y* = Bottom + (Top − Bottom)/(1 + 10^∧^((LogED50-X) × Hill  slope)) [25]. In order to generate isobolograms [25] the ED90 values of the drug combination and the drug alone were calculated from the linear equation of the dose-response curve of the drug alone and in combination 7 days after treatment had ended. The ED90 values were then used to calculate the isobolar equivalent (IE) values [25, 26]. Isobolograms were designed to include a diagonal line (black solid) which represents the line of additivity (Figures 2 and 4). Isobolograms allowed the visualization of additivity, synergism, or antagonism. If the IE values are below 1 it produces an isobologram that skews below the additivity line indicating synergism [25, 26]. When the IE values are equal or close to 1 most values will lie closely to the additivity line indicating additivity. If most IE values are above 1, this indicates antagonism [25, 26].

### 2.5. Statistical Analysis

A student *t*-test and the ANOVA test were used for statistical analysis using GraphPad Prism 4 software and SPSS software version 9.0 USA. All experiments were carried out according to the guidelines of the animal facility of the Institute of Hygiene and Tropical Medicine (IHMT), Lisbon, Portugal and according to the FELASA guidelines. 

### 2.6. Expression Profile of Genes Encoding Enzymes of the Ubiquitin/Proteasome Pathway in *P. chabaudi*

As previously mentioned curcumin is known to interfere with the UPS [14]. This was verified here by RT-PCR. In this study we selected three genes encoding deubiquitylating enzymes and the sequences were retrieved from the PlasmoDB database and used for primer design (Table 2). The human homologue of *PcUCH-L3* regulates the apical membrane recycling of epithelial sodium channels [31]. The human homologue of *PcUCH-L5* appears to be associated with the proteosome and possibly involved in TG*β* signaling [19]. UBP-8 gene in yeast regulates transcription mechanisms and it is responsible for the deubiquitylation of histone H2B [32]. Given the importance of these genes in other biological systems, we analyzed the basal expression of these genes in *P. chabaudi* throughout the parasite's life cycle and their response to treatment with subtherapeutic doses of chloroquine (2 mg/kg), artemisinin (2 mg/kg), and curcumin (2 mg/kg). 

### 2.7. *Plasmodium chabaudi* RNA Extraction and cDNA Synthesis

*Plasmodium chabaudi*-infected red blood cells were collected at time point 0 h; a microscopy analysis revealed that the parasites present were mainly young trophozoites. Infected red blood cells were passed through a column of fibrous cellulose powder (CF11) (Sigma-Aldrich, Madrid, Spain) to remove lymphocytes. The resulting solution was centrifuged at 700 g for 5 minutes and RNA was extracted from the obtained pellet using Trizol (Sigma-Aldrich, Madrid, Spain) and following the manufacturer's instructions. RNA (1 *μ*g) was mixed with 5 *μ*L of DNase I buffer and 1 *μ*L of DNase I (Promega, Mannheim, Germany) and incubated at 37°C for 15 mins. DNaseI was inactivated by adding 1 *μ*L of EDTA (Promega, Mannheim, Germany) and incubated at 65°C for 5 min. RNA (50 ng) previously treated with DNase I was used as template and was mixed with the Maxima first strand cDNA synthesis kit for RT-PCR (Fermentas, Madrid, Spain) and water to a final volume of 25 *μ*L according to the manufacturer's instructions. Samples were incubated for 10 min at 25°C, followed by 30 min at 50°C and the reaction of cDNA synthesis was terminated by incubation at 85°C for 5 min.

### 2.8. Real-Time PCR Analysis of *P. chabaudi* Genes Encoding Deubiquitylating Enzymes and RT-PCR Conditions

Real-time PCR using the IQ SYBR green supermix (Bio-RAD, Lisbon, Portugal) was carried out using microAmp 96-well plates (Applied Biosystems, Madrid, Spain) in triplicates with a 25 *μ*L final volume containing IQ SYBR green supermix dye, 0.025 U/*μ*L iTaq DNA polymerase (Promega, Mannheim, Germany), 200 *μ*M dNTPs (Promega, Mannheim, Germany), and 3,5 mM MgCl_2_ (Promega, Mannheim, Germany), and each individual mixture contained 300 nM of the forward primer (StabVida, Lisbon, Portugal) and 300 nM (StabVida, Lisbon, Portugal) of the reverse primers specific for *PcUCH-L3*, *PcUCH-L5*, and *PcUBP-8*; finally, 2 *μ*L of cDNA diluted 1 : 10 obtained from infected mice treated with curcumin, chloroquine, and artemisinin were added to each individual mixture. 

The reaction was performed under the following amplification conditions: 10 min of preincubation at 95°C, followed by 40 cycles for 15 seconds at 95°C and 1 min at 60°C in a 7500 fast RT-PCR thermocycler (Applied Biosystems, Madrid, Spain). Primers designed against *P. chabaudi Pc*β*-actinI* gene (*Pc*β*-actinI*) were used as the endogenous control as previously reported by others [33]. In order to determine PCR efficiencies for each individual gene, samples were diluted in serial 10-fold ranges, and the CT value at each dilution was measured. 

A curve was then constructed for each gene from which efficiency was determined. Real-time PCR efficiencies (*E*) were calculated from given slopes according to the equation: *E* = 10(−1/slope), where *E* = 2 corresponds to 100% efficiency [34].

### 2.9. Analysis of Relative Expression Using the 2^−ΔΔCT^ Method

The 2^−ΔΔCT^ method was used to calculate the relative quantification of target gene [34]. The purpose was to analyze first the expression of the studied genes throughout the parasite's life cycle, in the absence of any kind of treatment. The second aim was to analyze the differences in expression of each studied gene in parasites exposed to drug treatment versus a sample collected at time 0 h which was not exposed to drug treatment. Differences in N-fold expression compared to the untreated sample were analyzed using a student *t*-test *P* ≤ 0.05 (*n* = 3 assays) performed in GraphPad Prism 4 Software and SPSS software version 9.0.

## 3. Results

### 3.1. *In Vivo* Efficacy and Drug Interaction Studies of Curcumin Alone and Curcumin/Piperine/Chloroquine and Curcumin/Piperine/Artemisinin Drug Combinations

Given the emergence of artemisinin combination therapy (ACT), drug resistance new antimalarials are urgently needed. The aim of this study was to determine the *in vivo* efficacy of curcumin alone or in combination: curcumin/piperine/chloroquine and curcumin/piperine/artemisinin in order to clarify their drug interactions in *Plasmodium chabaudi* resistant parasites.

**Figure 1:**
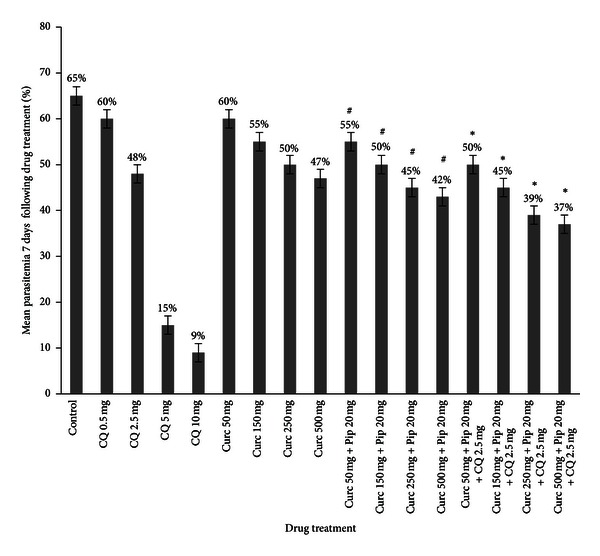
Parasitamia evolution in mice infected with *P. chabaudi* clone AS-3CQ treated with: chloroquine, curcumin, curcumin/piperine, and curcumin/piperine/chloroquine. Drugs were administered by oral gavage for 4 consecutive days and parasitemia was followed every day following drug treatment for 7 consecutive days. Results represent the mean parasitemia ± S.D. ^#^Significant difference (*P* < 0.05) was found between the curcumin/piperine treatment group versus the curcumin only treatment group. *Significant difference (*P* < 0.05) was found between curcumin/piperine/chloroquine group versus the curcumin only treatment group, curcumin/piperine treatment group, and chloroquine only treatment group (2.5 mg/kg).

The acute toxicity studies revealed that curcumin was nontoxic to mice even at 2 g/kg/bw (Table 1); mice survived for 14 days with no signs of toxicity. The results show that curcumin alone was able to delay peak parasitemia in a dose-dependent manner (Figures 1 and 3) in both *P. chabaudi* clones. Statistically, there was no significant difference (*P* > 0.05) between the control untreated group and the groups treated with 50 mg and 150 mg curcumin alone. Significant results were observed especially at 500 mg/kg/bw where parasitemia dropped to 47% in the AS-3CQ clone and 45% in the AS-ART clone compared to the control untreated group (*P* = 0.003) 65% and 62% (Figures 1 and 3).

Curcumin combined with piperine showed a mild antimalarial effect which is in agreement with previous work [7]. Again in both clones curcumin/piperine combination was more efficient at reducing parasitemia at higher doses. At 250 mg curcumin + 20 mg of piperine parasitemia dropped to 45% in mice infected with the chloroquine resistance clone (AS-3CQ) and 44% in mice infected with the artemisinin resistance clone (AS-ART) relative to the control (*P* = 0.04) (Figure 3). When curcumin at 500 mg was combined with 20 mg of piperine parasitemia dropped to 42% in mice infected with the chloroquine-resistant parasite line and 40% in mice infected with the artemisinin-resistant parasite line with *P* value significant (*P* = 0.02), indicating that the efficacy of the curcumin/piperine combination in *P. chabaudi* clones was also in a dose-dependent manner. For the drug interaction studies four doses of chloroquine were administered orally to mice infected with the chloroquine-resistant parasite line (AS-3CQ) and of the 4 doses given 2,5 mg of chloroquine was found to reduce parasitemia to 48% after 7 days post drug treatment. AS-3CQ parasites were treated with 5 mg/kg and 10 mg/kg which reduced parasitemia to 15% and 9%, respectively. Hence a choice was made not to combine these higher doses with curcumin/piperine as they would mask the effect of the combination.

When curcumin/piperine was combined with a fixed dose of 2,5 mg/kg of chloroquine, parasitemia reduction was better than when curcumin was used either alone or when it was used in combination with piperine (Figure 1). When curcumin/piperine/chloroquine was administered to mice a significant reduction in parasitemia was achieved compared to the control group. This reduction was even more evident at higher doses (150 mg + 20 mg + 2,5 mg) parasitemia 45% (*P* = 0.033), (250 mg + 20 mg + 2,5 mg) parasitemia 39% (*P* = 0.01), and (500 mg + 20 mg + 2,5 mg) parasitemia 37% (*P* = 0.0001) relative to the control sample (Figure 1).

**Figure 2:**
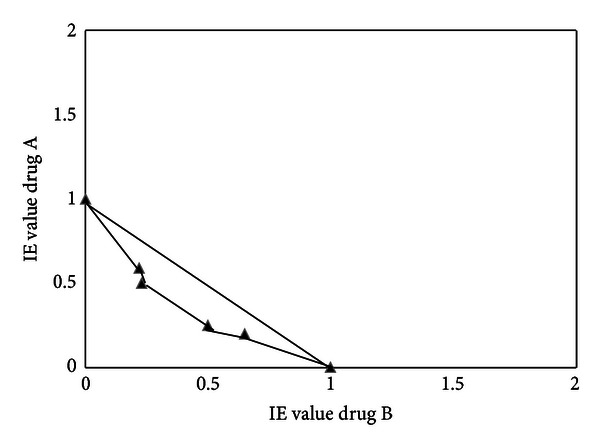
Isobologram illustrating the *in vivo* interaction at the ED90 level between drug A (curcumin-piperine) with drug B (chloroquine) in *Plasmodium chabaudi* AS-3CQ chloroquine-resistant parasites. Plotting the percentage parasitemia inhibition versus the log dose of the drugs either alone and the drugs in combination using GraphPad Prism 4 software yielded a regression equation. The equation allowed the determination of the ED90 of the drugs alone and the drugs in combination. ED90 values were used to obtain isobolar equivalents (IE) [25, 26].

It is interesting to note that when curcumin/piperine/chloroquine are combined together less curcumin is needed to actually see a statistically significant suppression in parasitemia. This is evident as parasitemia dropped to 50% with the combination (curc 50 mg + pip 20 mg + CQ 2,5 mg) (*P* < 0.03) compared to the control group 65%, indicating an additive/weak synergistic effect which was confirmed by the isobologram (Figure 2) with most values achieved below 1,5. Although the interaction of curcumin/piperine/chloroquine was favorable and helped to reduce the parasite load, a followup of parasitemia for another 8 days (total 15 days) showed that a complete clearance of parasites to submicroscopic levels was not achieved. There was always a residual parasitemia of <2% detected in the slides. An indication that the parasites did not lose their chloroquine-resistant phenotype.

**Figure 3:**
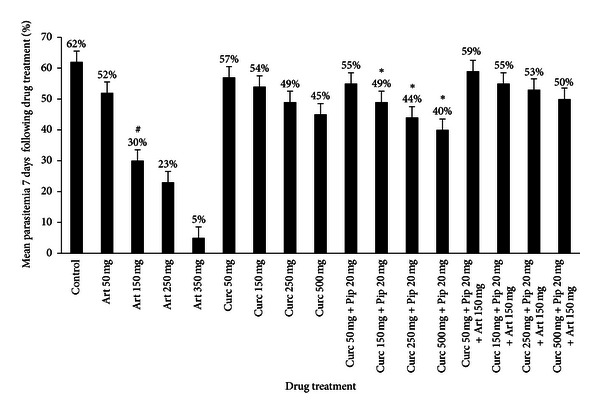
Parasitemia evolution in mice infected with *P. chabaudi* clone AS-ART treated with: artemisinin, curcumin, curcumin/piperine, and curcumin/piperine/artemisinin. Drugs were administered by oral gavage for 4 consecutive days and parasitemia was followed every day following drug treatment for 7 consecutive days. Results represent the mean parasitemia ± S.D. ^#^Significant difference was found between the artemisinin only treatment group (150 mg/kg) versus the curcumin, curcumin/piperine, and curcumin/piperine/artemisinin. *Significant difference was found (*P* < 0.05) between the curcumin/piperine group versus the curcumin only treatment group and the curcumin/piperine/chloroquine.

The group of mice infected with the artemisinin-resistant parasite line AS-ART was also treated with four doses of artemisinin alone (50 mg, 150 mg, 250 mg, and 350 mg) and at 350 mg/kg of artemisinin alone parasitemia dropped to 5% compared to the control group 67% (*P* = 0.0001) (Figure 3). A decrease in parasitemia was also observed at 50 mg, 150 mg, and 250 mg (Figure 3) and 150 mg/kg was chosen as the dose used to combine with curcumin/piperine (Figure 3).

Again treatment of *Plasmodium chabaudi* resistant AS-ART parasite line with curcumin alone only resulted in a significant parasitemia reduction at higher doses. At 500 mg/kg of curcumin parasitemia reduced to 45% (*P* = 0.0001) compared to the control group 62% (Figure 3). This reduction was even more evident when curcumin was combined with piperine which resulted in a parasitemia reduction from 67% to 40% (*P* = 0.0001) (Figure 3).

Addition of a fixed dose of 150 mg/kg of artemisinin to curcumin/piperine did not result in a clear difference in parasitemia reduction between the control group and the artemisinin/curcumin/piperine (*P* = 0.08) which is in agreement with previous work [7]. In fact, even at higher doses (curcumin 500 mg + piperine 20 mg + 150 mg artemisinin) parasitemia dropped to 50% relative to the control group (*P* = 0.055) 62% (Figure 3).

**Figure 4:**
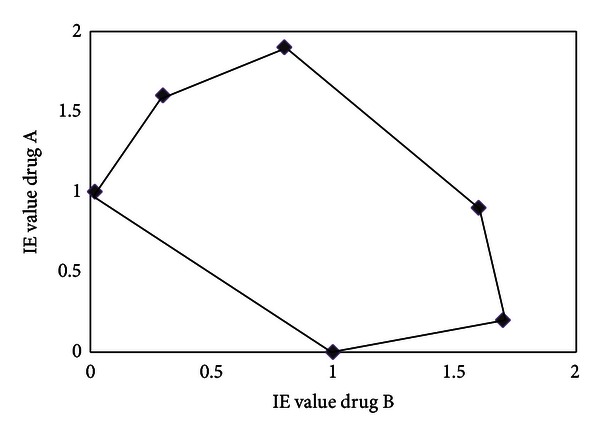
Isobologram illustrating the *in vivo* interaction at the ED90 level between drug A (curcumin-piperine) with drug B (artemisinin) in *Plasmodium chabaudi* AS-ART (artemisinin) resistant parasites. Plotting the percentage parasitemia inhibition versus the log dose of the drugs either alone and the drugs in combination using GraphPad Prism 4 Software yielded a regression equation. The equation allowed the determination of the ED90 of the drugs alone and the drugs in combination. ED90 values were used to obtain isobolar equivalents (IE) [25, 26].

It seems that curcumin, curcumin/piperine, and artemisinin alone performed better separately as opposed to when the three compounds are combined (Figure 4). The isobologram indicates that most values were bigger than 1,5 which resulted in an isobologram where most of the values are far away from the additivity line, indicating antagonism amongst the components of the drug combination. 

### 3.2. Expression of the Genes *PcUCH-L3*, *PcUCH-L5*, and *PcUBP-8* in *P. chabaudi* Parasites and the Effects of Drug Treatment on the Expression of Those Genes

**Figure 5:**
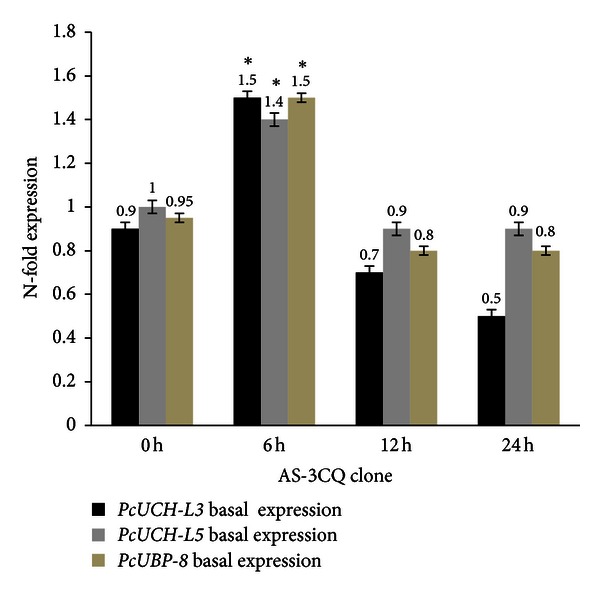
Basal expression profile of the genes *PcUCH-L3*,* PcUCH-L5*,* and UBP-8* in the clone AS-3CQ. The results are expressed as means and standard deviation of three independent experiments. *Indicates significant difference (*P* < 0.05) between the sample collected at time point 0 h versus 6 h samples. Statistically no significant (*P* > 0.05) difference was found between the samples collected at time point 12 h and 24 h sample versus the 0 h sample.

**Figure 6:**
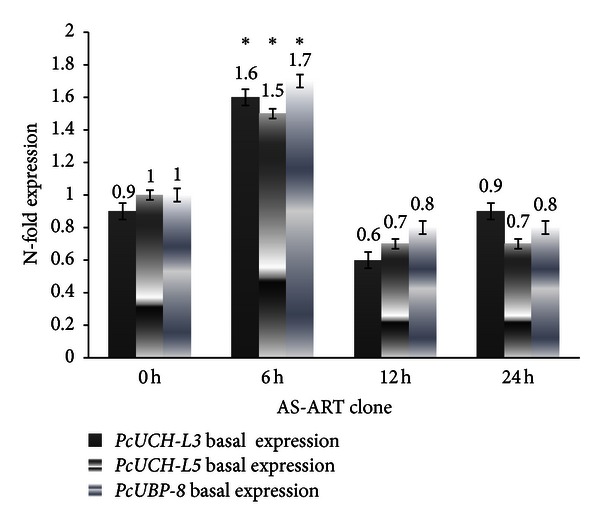
Basal expression profile of the genes *PcUCH-L3*, *PcUCH-L5*,* and UBP-8* in the clone AS-ART. The results are expressed as means and standard deviation of three independent experiments. *Indicates significant difference between (*P* < 0.05) between the sample collected at time point 0 h versus 6 h samples. Statistically no significant (*P* > 0.05) difference was found between the samples collected at 12 h and 24 h versus 0 h.

Three genes encoding de-ubiquitylating enzymes *PcUCH-L3*, *PcUCH-L5,* and *PcUBP-8* were analyzed by RT-PCR in the presence and absence of drug treatment in order to verify whether drug treatment interferes with the UPS. The results suggest that in both clones AS-3CQ and AS-ART the three genes under study are highly expressed at 6 h time point (Figures 5 and 6). Microscopic slides prepared at that time point when mRNA was collected revealed most of the parasites were mainly at the mature trophozoite stage, which is the most replicative stage of the parasite's life [35], and statistically there is no difference in the basal expression of the three genes between the two clones (*P* = 0.08).

**Figure 7:**
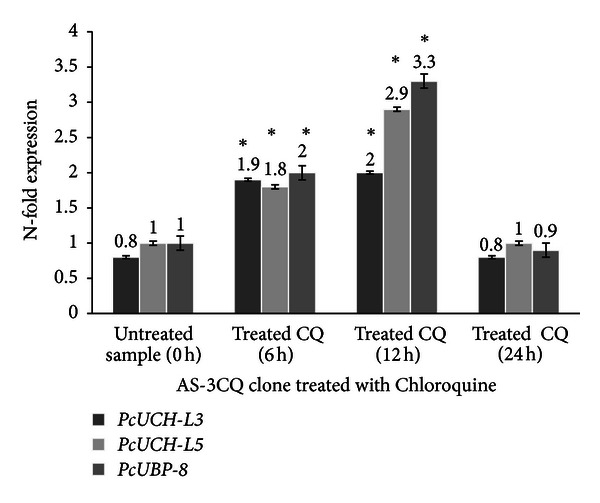
Expression profile of the genes *PcUCH-L3*, *PcUCH-L5*,* and UBP-8* exposed to chloroquine treatment. The results are expressed as means and standard deviation of three independent experiments. *Indicates significant difference (*P* < 0.05) between the sample collected at time point 0 h versus 6 h samples and the 12 h sample. Statistically no significant (*P* > 0.05) difference was found between the samples collected at time point 0 h versus 24 h sample.

**Figure 8:**
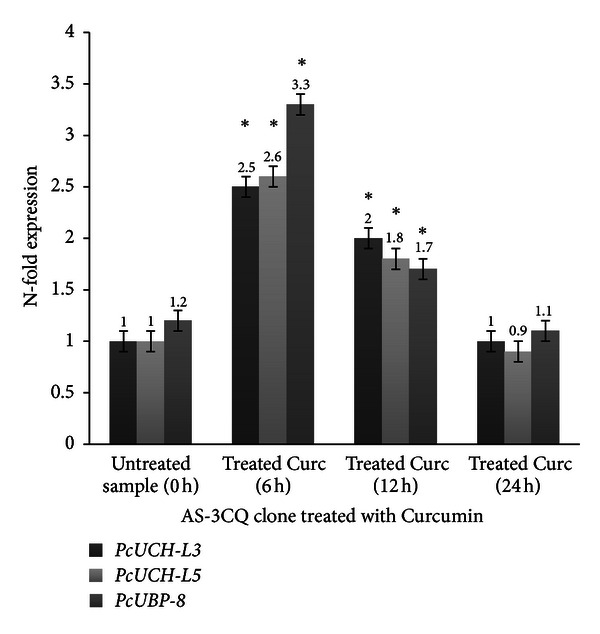
Expression profile of the genes *PcUCH-L3*, *PcUCH-L5*,* and UBP-8* exposed to curcumin treatment. The results are expressed as means and standard deviation of three independent experiments. *Indicates significant difference (*P* < 0.05) between the sample collected at time point untreated 0 h versus 6 h samples and the 12 h sample. Statistically no significant (*P* > 0.05) difference was found between the samples collected at time point (0 h) versus 24 h sample.

Treatment of AS-3CQ parasites with a subcurative single dose of chloroquine and a subcurative single dose of curcumin induced an increase in the expression of all three genes (Figures 7 and 8) specially at 6 h and 12 h after drug exposure, compared to the untreated sample collected at time point 0 h (*P* = 0.002). Statistical analysis was also applied to compare the basal expression of the genes at 6 h and 12 h time points versus the same samples exposed to treatment, which revealed a difference (*P* = 0.01). Microscopic slides analyzed revealed mostly the presence of schizonts at 12 h time point, which is also the stage of the parasite in which chloroquine is known to act. The effects appear to be transient with all genes returning to levels similar to the untreated sample 24 hours later. Microscopic slides revealed the presence of ring-stage parasites at the 24 h collection point. In both treatments *PcUBP-8* was the gene that exhibited the highest increase in expression relative to the untreated sample 0 h (*P* = 0.001) (Figures 7 and 8). 

**Figure 9:**
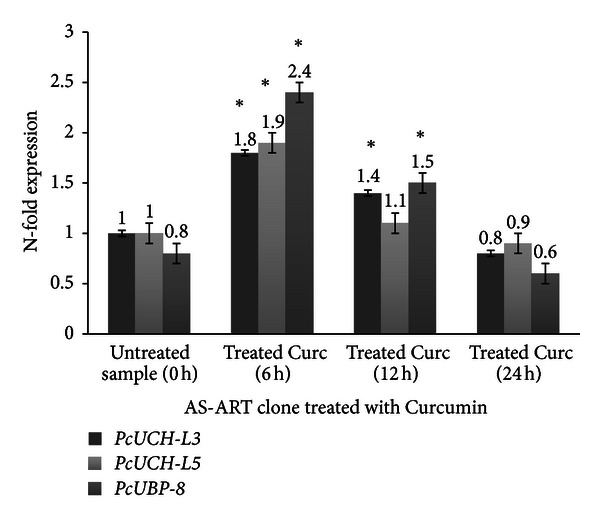
Expression profile of the genes *PcUCH-L3*, *PcUCH-L5*,* and UBP-8* in the clone AS-ART exposed to curcumin treatment. The results are expressed as means and standard deviation of three independent experiments. *Indicates significant difference (*P* < 0.05) between the sample collected at time point untreated 0 h versus 6 h samples and the 12 h sample. Statistically no significant (*P* > 0.05) was found between the samples collected at time point 0 h versus 24 h sample.

**Figure 10:**
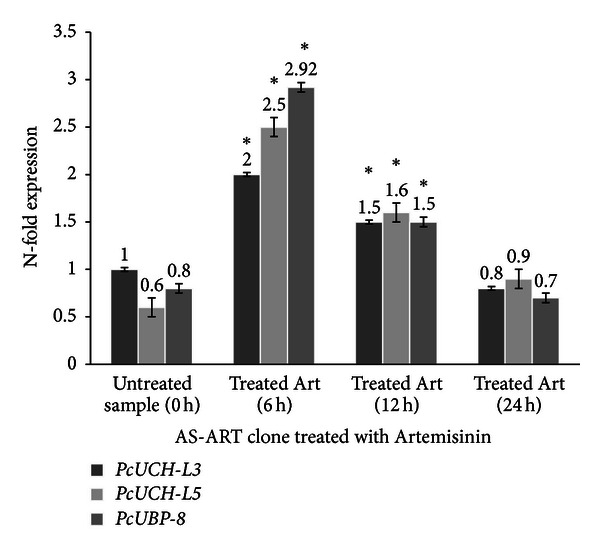
Expression profile of the genes *PcUCH-L3*, *PcUCH-L5*,* and UBP-8* in the clone AS-ART exposed to artemisinin treatment. The results are expressed as means and standard deviation of three independent experiments. *Indicates significant difference (*P* < 0.05) between the sample collected at time point 0 h versus 6 h samples and the 12 h sample. Statistically no significant (*P* > 0.05) difference was found between the samples collected at time point 0 h versus 24 h sample.

In AS-ART parasites treated with a single subcurative dose of artemisinin and a single subcurative dose of curcumin there was also an increase in the expression of all three genes relative to the untreated sample 0 h, which was more evident at 6 h and 12 h following drug exposure (*P* = 0.002). In both treatments *PcUBP-8* was the gene that exhibited the highest increase in N-fold expression relative to the control untreated sample (*P* = 0.0001). The expression levels induced by drug treatment were transient with the three genes returning to levels similar to the untreated sample 0 h (Figures 9 and 10). 

## 4. Discussion

Given the emergence of drug resistance against ACTs, new alternatives for the treatment of malaria are urgently needed. Curcumin has already shown great potential both *in vitro* and *in vivo* against *Plasmodium *spp. [6, 7]. However, its poor availability and rapid metabolism are issues to overcome in order to exploit the full benefits of this plant-derived compound [36]. Enhancers such as piperine derived from black pepper which is already known to improve the bioavailability of curcumin [36] were hereby tested as a combination: curcumin/piperine/chloroquine and curcumin/piperine/artemisinin. 

The results show that the interaction between curcumin/piperine/chloroquine was additive and helped in the reduction of the parasite load 7 days after treatment had ended. The results are interesting: although both drugs have different structures and different modes of action, they both have anti-inflammatory properties which possibly contribute to parasitemia reduction [37]. Curcumin is well known for its immunomodulatory properties which include: activation of TLR2, increase in IL-10, and production of antiparasite antibodies [37]. Chloroquine is well known for its antimalarial schizonticidal activity as well as its anti-inflammatory properties such as inhibition of tumor necrosis factor-*α*, IL-1*β*, and IL-6 [38] making both drug combinations interesting in the treatment of other diseases where an excess of proinflammatory cytokines is produced. It is believed that curcumin is an attractive compound for adjunctive treatment of cerebral malaria [39] which is often treated with quinine, from which chloroquine derives. Hence further pharmacokinetic studies between curcumin and quinine and its derivatives are needed to exploit their potential in antimalarial treatment.

The combination of curcumin/piperine/artemisinin did not show a favourable drug interaction. Although it was able to reduce parasitemia statistically there was no difference between the control untreated group and the curcumin/piperine/artemisinin group. In fact the mice treated with artemisinin alone and curcumin alone actually had a significant reduction in parasitemia compared with mice treated with the drug combination curcumin/piperine/artemisinin (Figure 3). 

An* in vitro* study carried out in *P. falciparum* revealed synergism between curcumin and artemisinin, and the *in vivo* assay where *α*,*β*-arteether was injected intramuscularly in *P. berghei*-infected mice followed by curcumin feeding was able to prevent recrudescence [6]. However, in the study mentioned piperine was not used as an enhancer and the difference in the biology of* P. chabaudi* and *P. berghei* parasites also needs to be taken into consideration. In the present study the mixture of the three compounds administered orally resulted in an unfavorable pharmacodynamic interaction. Recent studies in *P*. *berghei*-infected mice using a combination of artemisinin and curcumin have also shown that although parasites can be cleared from the blood, they remain in the spleen and the liver [37], favoring recrudescence. 

Artemisinin has a half life of approximately 8–14 hours [37] and curcumin has a half life of 8 h [37]. Studies carried out in rats have reported that only about 0,1%–0,25% of piperine administered orally can be detected in the liver whereas intraperitoneal administration of piperine resulted in 1–2,5% of piperine detection in the liver 6 hours later [40]. Given the fact that the three drugs have different structures and different modes of action, clearly more studies are needed including different administration routes and HPLC analysis of mice tissue after treatment with curcumin/piperine/artemisinin in order to clarify drug distribution and elimination.

Artemisinin derivatives as well as curcumin derivatives might offer an alternative to the pure compound. A Study has shown that the pyrazole analogue of curcumin (methyl curcumin) has 7–9 fold activity against *P. falciparum* CQ-sensitive and CQ-resistant strains with IC50 values of 0.48 and 0.45 *μ*M [41] compared to curcumin which has an *in vitro* IC50 of 5 *μ*M [41]. Previous work has also shown that artemisinin at 50 mg/kg/bw combined with curcumin at 100 mg/kg/bw encapsulated in conventional liposomes cured all *P. berghei* malaria-infected mice when compared to free drug [42]. This study concludes that delivery systems such as liposomes and nanoparticles may be the key in delivering drugs with short half life and poor bioavailability [42]. It has also been shown that curcumin when bound to chitosan nanoparticles is able to completely cure *P. yoelii* infected mice [43]. Hence future studies with curcumin should aim at finding suitable drug-delivery systems [43] and better drug partners in order to maximize the benefits of curcumin as an antimalarial agent.

The gene expression assays showed evidence of an active UPS in *P. chabaudi* parasites mainly at the trophozoite stage which coincides with high metabolic activity, which is necessary for parasite replication [35]. In the present study all three drugs administered orally were able to induce transient changes in the expression of the three genes with *PcUBP-8* showing the highest increase when exposed to all three drugs (Figures 7, 8, 9, and 10) relative to the untreated sample. As mentioned previously, *PcUBP*-*8* gene in yeast *Saccharomyces cerevisiae* is required for optimal gene activation and is responsible for the de-ubiquitylation of histone H2B, which is involved in chromatin remodeling [32]. In *P. chabaudi* parasites the role of *PcUCH-L3*, *PcUCH-L5*, and *PcUBP-8* enzymes is unknown. It is not clear whether these enzymes are controlling epigenetic mechanisms or regulating the transcription of *P. chabaudi* genes. Previous work using serial analyses of gene expression (SAGE) technique has shown that in chloroquine-treated *P. falciparum* cultures a 5.5-fold increase in a gene encoding an ubiquitin-specific protease and a 5.5-fold increase in a gene encoding a proteosome subunit *α* were observed [44]. On the other hand, treatment of human breast carcinoma MCF-7 cells with doxorubicin, which also has antimalarial activity [45], showed an 18.6-fold increase in the 26S proteosome regulatory subunit [46] indicating that alterations in the UPS may represent a general adaptation of the parasite to drug treatment.

It is already known that drug treatment can interfere with ion homeostasis, which would result in alterations in the intracellular pH of several organelles that are crucial for parasite survival, thereby interfering with enzyme activity and function [47], which would explain the necessity of an increase in the transcription and translation of *PcUCH-L3*, *PcUCH-L5*, and *PcUBP-8* gene products to compensate for enzyme damage. Damaged proteins are likely to act as a stimulus to induce the expression of genes encoding enzymes involved in protein synthesis and protein degradation in order to allow the parasite to cope with intracellular stress. Hence, upregulation of the UPS in general would be needed to help the parasite survive under drug pressure, which could very well be a mechanism of defense and or resistance, making the UPS an attractive drug target [48].

## Figures and Tables

**Table 1:** Acute toxicity test for curcumin. Mice were allowed to fast 4 hours. After 4 hours a single dose of curcumin was orally administered in different concentrations to BALB/c infected mice. Mice were observed for 14 days for any physical signs of toxicity.

LD50 cytotoxicity test	Weight	Survival (days)
2,0 g per kg of body weight	15 g	14 days
2,5 per kg of body weight	15 g	10 days
3,0 g per kg of body weight	15 g	5 days
3,5 g per kg of body weight	15 g	3 days
5,0 g per kg of body weight	15 g	1 day

**Table 2:** Oligonucleotide primers designed from the mRNA sequences retrieved from PlasmoDB.

Oligonucleotide primers		Primer	Sequence	Amplicon size (bps)
Gene ID	Gene		
PCHAS-146160	*Pc-B-actin I *	Forward Reverse	GCAATGTATGTAGCAATTCA GCATGGGGTAATGCATAACC	131
PCHAS-091340	*PcUCH-L5 *	Forward Reverse	AAATGCTGAAGCAGATGGGCG GGTTCTGTCCCCATTTCTGCTT	181
PCHAS-132740	*PcUCH-L3 *	Forward Reverse	CGGGAAGTGATTTAAATGCAG GCACTTGTGGTTTGGCCATGG	136
PCHAS-041770	*PcUBP8 *	Forward Reverse	GGCGGTCGAAATACCTCTAACC CATCATTACTGTTGGATTGGCTC	127
